# Research on surface treatment technology for quickly improving the skid resistance of tunnel concrete pavement

**DOI:** 10.1371/journal.pone.0295938

**Published:** 2024-03-11

**Authors:** Jun’an Lei, Fujing Zhao, Yuanyuan Wang, Xiaofeng Ren

**Affiliations:** 1 School of Civil Engineering and Architecture, HuBei University of Arts and Science, Xiangyang, Hubei, China; 2 School of Education, HuBei University of Arts and Science, Xiangyang, Hubei, China; 3 Xiangyang Road and Bridge Construction Group Co., Ltd., Xiangyang, Hubei, China; Texas A&M University System, QATAR

## Abstract

In order to solve the problem that the skid resistance of concrete pavement in tunnel deteriorates rapidly, which is easy to cause traffic accidents, the anti-skid rapid elevation technology of surface treatment is proposed. Wear tests were used to investigate the effects of concrete surface roughness, properties of modified emulsified asphalt binder and anti-skid fine aggregate type on long-term skid resistance of treated surfaces. The results show that the four coarsening methods of fine milling, milling, grooving and brooming can improve the skid resistance of concrete, and the skid resistance durability of fine milling and milling is better. The adhesive property of modified emulsified asphalt is the best when the content of water-based epoxy resin is 20%. In different aggregates, the anti-skid effect is better when silicon carbide is used as anti-skid aggregate and the particle size is 0.6mm:0.3mm = 2:3. The method of fine milling of concrete surface + spraying epoxy emulsified asphalt + spreading silicon carbide can effectively improve the anti-skid performance of the original concrete pavement, and the feasibility of the scheme is verified by the test road. The research results have a good reference value for improving the skid resistance of tunnel concrete pavement.

## 1 Introduction

As roads extend towards mountainous and hilly areas, more and more tunnels have been built. Considering the requirements for environmental protection and fire safety, concrete was mostly used to construct the tunnel pavement [1]. Concrete has the advantages of high strength and stiffness, so structural diseases rarely occur on tunnel pavement. Due to the closed environment of the tunnel, car exhaust is prone to accumulate, air humidity is high, traffic channelization is severe, and braking is frequent, resulting in a sharp decline in the anti-skid performance of the tunnel concrete pavement [2, 3]. Generally, after 3 to 5 years of operation, the anti-skid performance of the road no longer meets the standard requirements, which is far shorter than the 30-year service life of the concrete pavement. This poses a great hazard to the driving safety [4]. Compared with ordinary roads, tunnel pavement materials have higher requirements for wear and skid resistance. Therefore, it is urgent to study the anti-skid treatment of tunnel concrete pavement.

In response to the problem of insufficient anti-skid performance of tunnel concrete pavement, exposed concrete technology can be used for newly built concrete pavement [5]. For old concrete pavement, regular cleaning with water can be used [6], but the most effective methods are the re-making anti-skid structures and the asphalt overlay. The methods of re-making anti-skid structures include manual chiseling, grooving [7, 8], and milling [9]. The overlay method includes laying sealing layer [10], micro-surfacing [11], Novachip overlay [12], and asphalt concrete ultra-thin overlay [13–15]. The anti-skid performance of the road surface is mainly determined by both macro and micro textures [16–18]. Therefore, the most effective way to improve the anti-skid performance is to simultaneously improve the macro [19] and micro [20] textures of the original concrete pavement [21]. The re-making anti-skid structures method only enhances the macro texture of the road surface, so the effect of this method on improving the anti-skid durability performance is not significant. Although the asphalt overlay method has some improvements in both macro and micro textures, it is not economical, and the construction process is complex and time-consuming.

**Fig 1:**
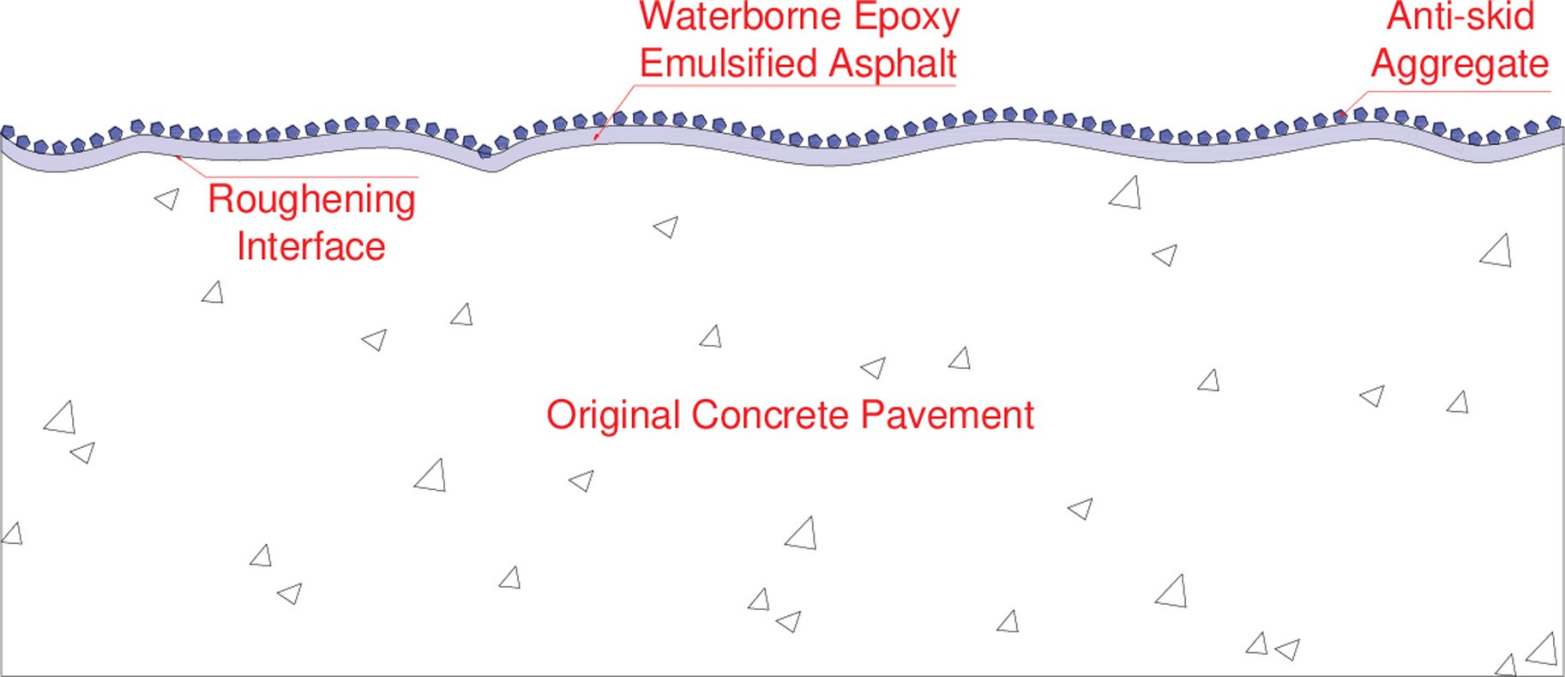
Principle of anti-skid treatment.

A tunnel concrete pavement surface treatment technology was proposed in this study to achieve the goal of quickly improving the skid resistance and durability of the pavement. The basic principle is that roughening technology is adopted to reshape the macro-texture of the original road surface, and then special binder and anti-skid aggregate are embedded in the road surface to enhance the micro-texture. The combined effect of macro-texture and micro-texture significantly improves the anti-skid performance of the original road surface, as shown in Fig 1. In order to improve the bonding strength between anti-skid aggregates and concrete pavement, water-based epoxy resin modified emulsified asphalt was selected as the bonding material, because of the good compatibility between water-based epoxy resin and emulsified asphalt. The influence of the roughness method and the type of anti-skid aggregate on the anti-skid durability performance of concrete pavement was studied. The reliability of the technology was verified through the paving and observation of experimental roads. The research results are meaningful for improving the anti-skid performance of tunnel concrete pavement and ensuring driving safety.

## 2 Materials

The materials selected for this study mainly include emulsified asphalt, epoxy resin, and anti-skid aggregate.

### 2.1 Emulsified asphalt

SBR modified emulsified asphalt has been selected, and its basic technical indicators are shown in Table 1.

**Table 1:** Technical indicators of emulsified asphalt.

Indicator		Unit	Value	Requirement	Test method
Remaining amount (1.18mm sieve)		%	0.003	≤0.1	T0652
Particle charge		-	Cation (+)	Cation (+)	T0653
Engla viscosity E25		-	5.8	1–10	T0622
Evaporation residue	Residue content	%	60.6	≥50	T0651
	Penetration (25°C)	0.1mm	61.7	40–120	T0604
	Softening point	°C	55.5	≥50	T0606
	Ductility (5°C)	cm	44.3	≥20	T0605

### 2.2 Water-based epoxy resin

According to the manufacturer’s instructions, the properties of water-based epoxy resin are shown in Table 2.

**Table 2:** Indicators of water-based epoxy resin.

Indicator		Component A	Component B
Appearance	Colour	Milky white	Light transparent
	State	Emulsion	Viscous liquid
Epoxy value (mol/100g)		0.068	—
Amine value (mol/100g)		—	0.515
Solid content (%)		49.48	38.35

### 2.3 Anti-skid aggregate

The selected anti-skid aggregates include silicon carbide, basalt, natural river sand, and limestone, with particle sizes of 0.3mm and 0.6mm. The technical indicators are shown in Table 3.

**Table 3:** Basic technical indicators of aggregates.

Aggregate type	Particle size (mm)	Density (g/cm^3^)	Crushing value (%)	Soundness(%)
Limestone	0.3	2.70	86.7	2.71
	0.6	2.71	83.2	
Basalt	0.3	2.99	97.3	2.39
	0.6	3.00	94.7	
River sand	0.3	2.75	94.2	2.82
	0.6	2.76	92.2	
Silicon carbide	0.3	3.51	94.8	1.29
	0.6	3.52	83.6	

## 3 Experimental

### 3.1 Performance test of modified emulsified asphalt binder

Water-based epoxy resin (A: B = 9:1) was added according to the quality of emulsified asphalt 0%, 10%, 20%, 30%, 40%, and then stirred by an electric mixer for 10 minutes to prepare water-based epoxy emulsified asphalt.

#### 3.1.1 Evaporation residue and three indexes

The evaporation residue of water-based epoxy emulsified asphalt was obtained according to ASTM D244, and then the penetration, softening point and 5°C ductility tests were conducted to analyze the influence of water-based epoxy resin on the high and low temperature performance of emulsified asphalt.

#### 3.1.2 Adhesion test

**Fig 2:**
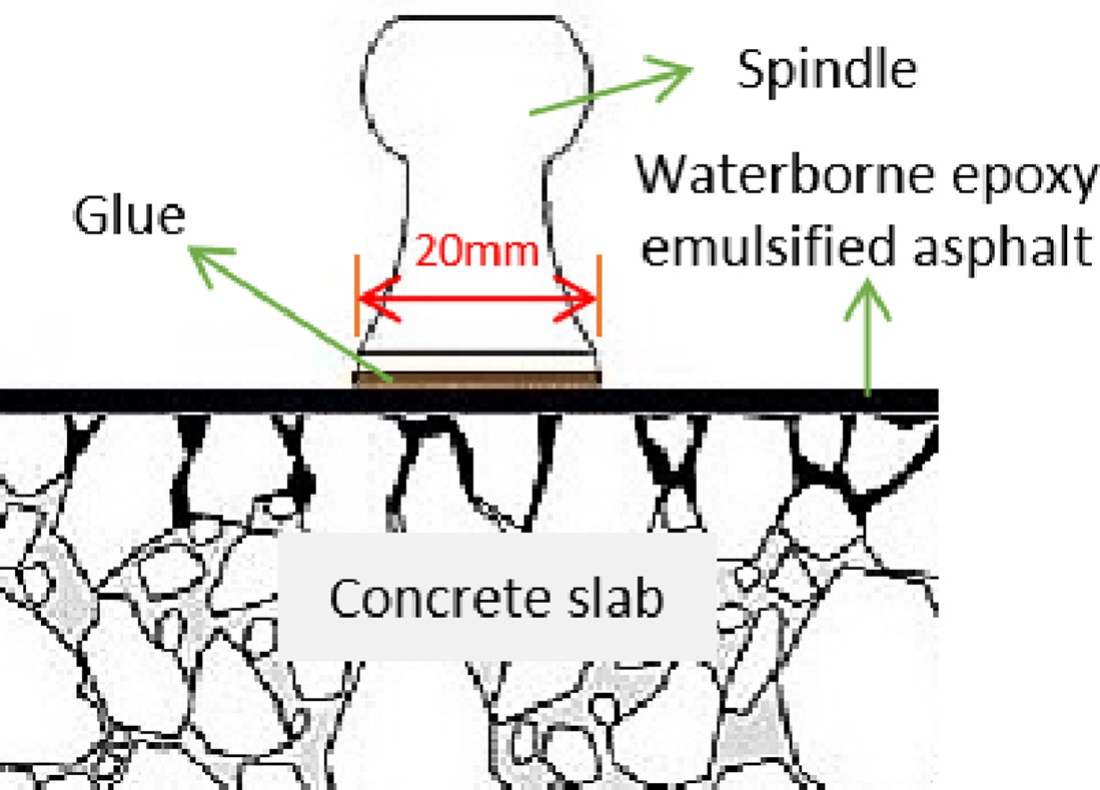
Adhesion test.

The adhesion is measured by using the PosiTest automatic pull-out tester, as shown in Fig 2.

The testing steps are: (1) The surface of the concrete slab was polished smooth and cleaned; (2) The water-based epoxy emulsified asphalt was brushed on the surface of the concrete slab with amount of 0.3kg/m^2^; (3) The glue was evenly applied to the surface of the spindle and then bonded to the slab. (4) After curing, the pull-out test can be conducted to measure the tensile strength. The test was divided into two working conditions: the first was conducted under the drying state at 25°C; the second was to place the sample in a 60°C water bath for 6 hours, and then take it out and cool it to 25°C for testing.

#### 3.1.3 Fluorescence microscopy test

**Fig 3:**
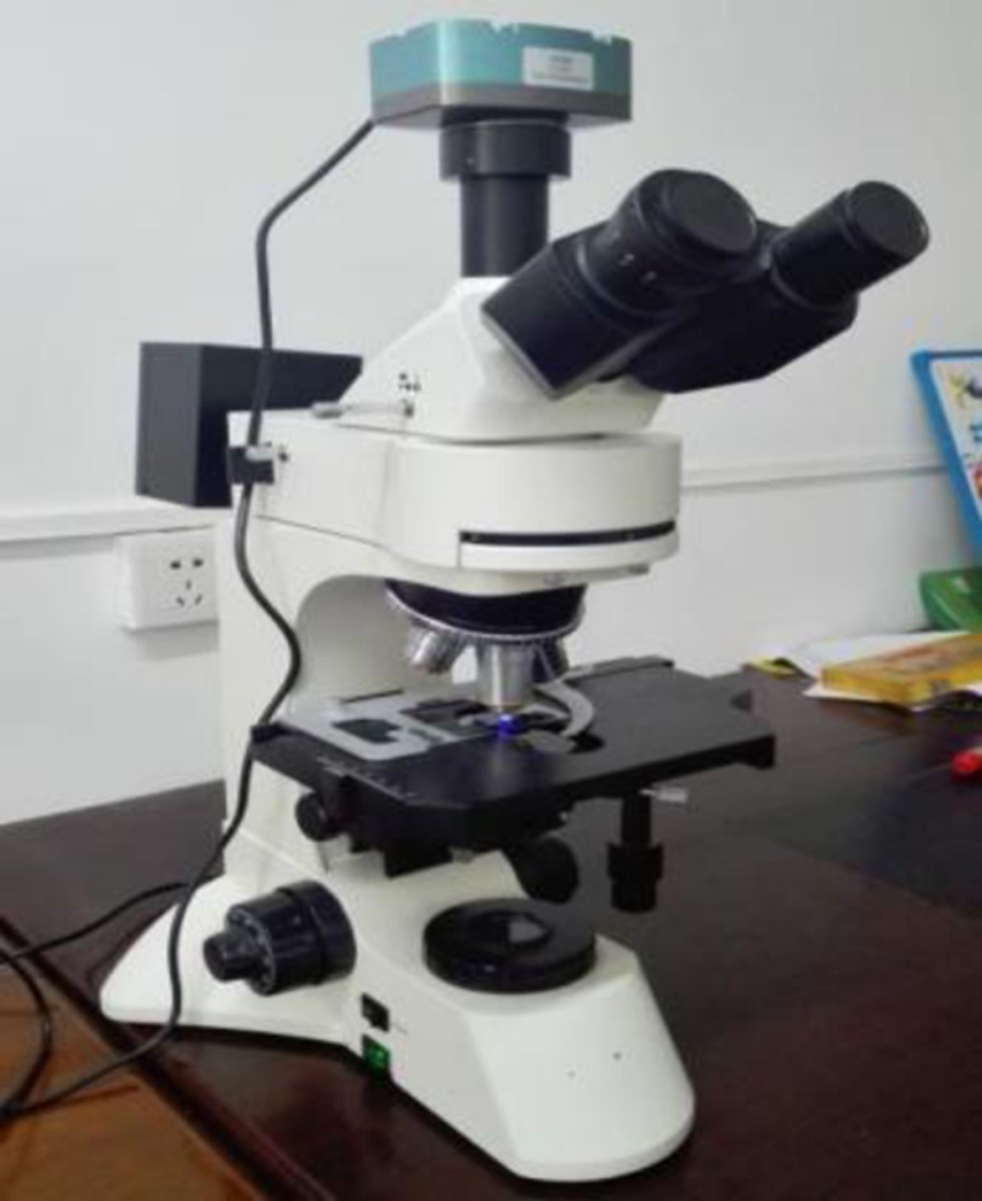
Fluorescence microscopy test.

The micro-morphology of water-based epoxy emulsified asphalt was observed and analyzed by using LW300LFT fluorescence microscope. An appropriate amount of the prepared water-based epoxy emulsified asphalt was dropped into the grooves of the single concave slide and smoothed, and the cover slide was gently covered. A 400x objective lens was used for this test image, as shown in Fig 3.

### 3.2 Surface roughness of concrete

A concrete slab with a size of 30cm×30cm×5cm was prepared in the test. Four treatment methods, including fine milling, milling, grooving and brooming, were selected to roughen the surface of concrete as the interface of water-based epoxy emulsified asphalt spreading. The roughening parameters are shown in Table 4.

**Table 4:** Roughening parameters.

Roughening method	Parameter	Machine
Fine milling	Depth 6mm	Small Wirtgen milling machine
Milling	Depth 2mm	Aishimu milling machine
Grooving	Depth 4mm, width 4mm, spacing 18mm	Artificial
Brooming	Depth 2mm	Artificial

**Fig 4:**
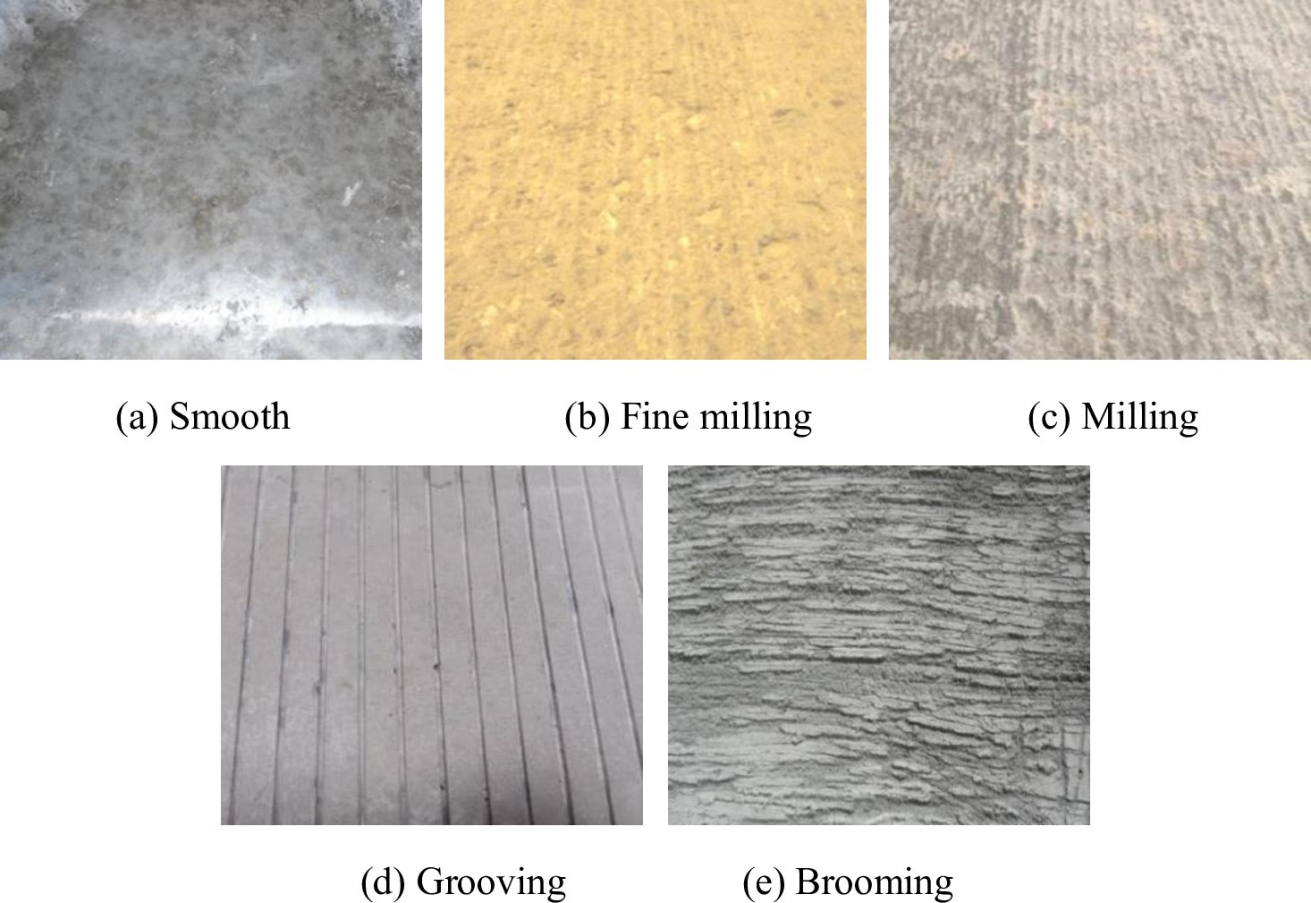
Roughened concrete surface. (a) Smooth, (b) Fine milling, (c) Milling, (d) Grooving, and (e) Brooming.

The roughened concrete surface is shown in Fig 4.

**Fig 5:**
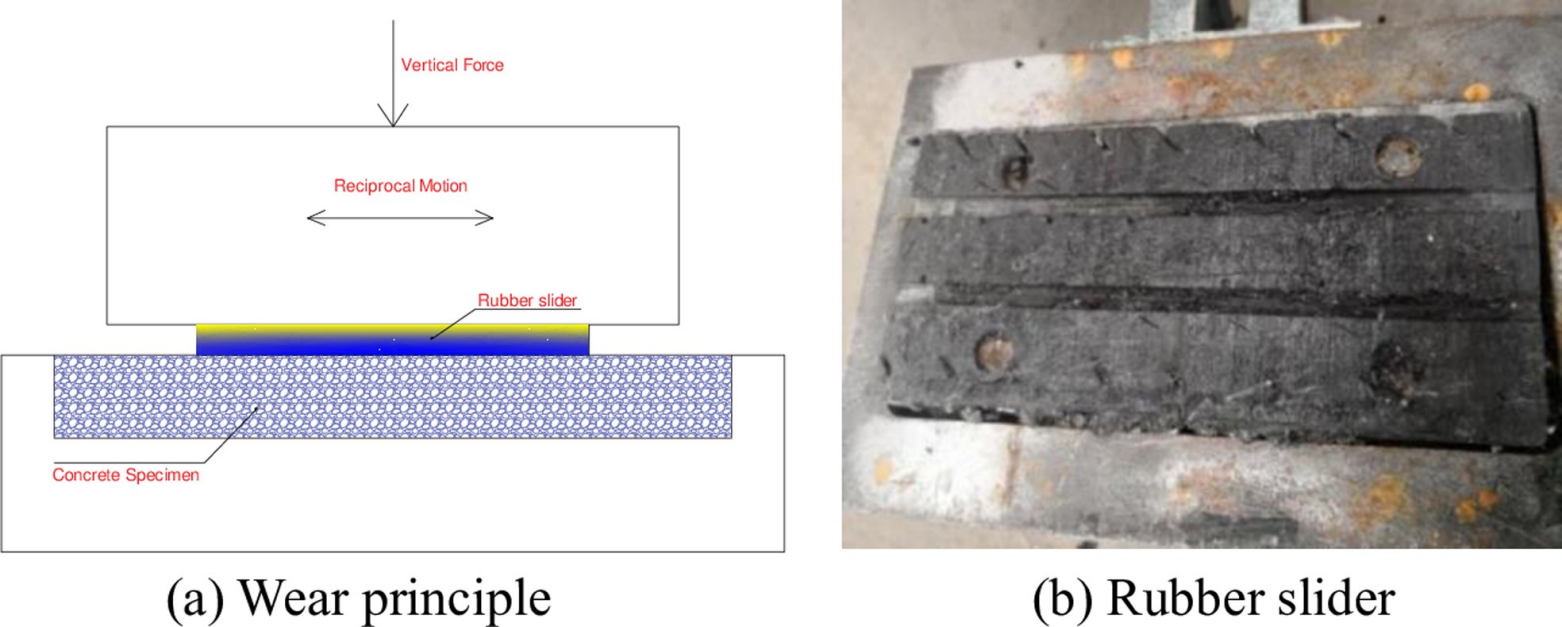
Wear test. (a) Wear principle and (b) Rubber slider.

In order to study the long-term anti-skid performance of concrete pavement with different treatment methods, a reciprocating wear tester was used to conduct abrasion test on the roughened surface, as shown in Fig 5. The concrete specimen was fixed on the lower side, and the rubber slider was used for horizontal round-trip movement on the upper side. The contact pressure was 0.7MPa, and the wear speed was 40 times /min. The number of wear was set as 0, 3000, 6000, 9000, 12000, 15000, 18000, 21000, and 24000 times.

After each wear, the pendulum friction tester and sand laying method were used to measure the anti-skid performance of the specimen, including British Pendulum Number (BPN) and Texture Depth.

### 3.3 Anti-skid performance of treated surface

**Fig 6:**
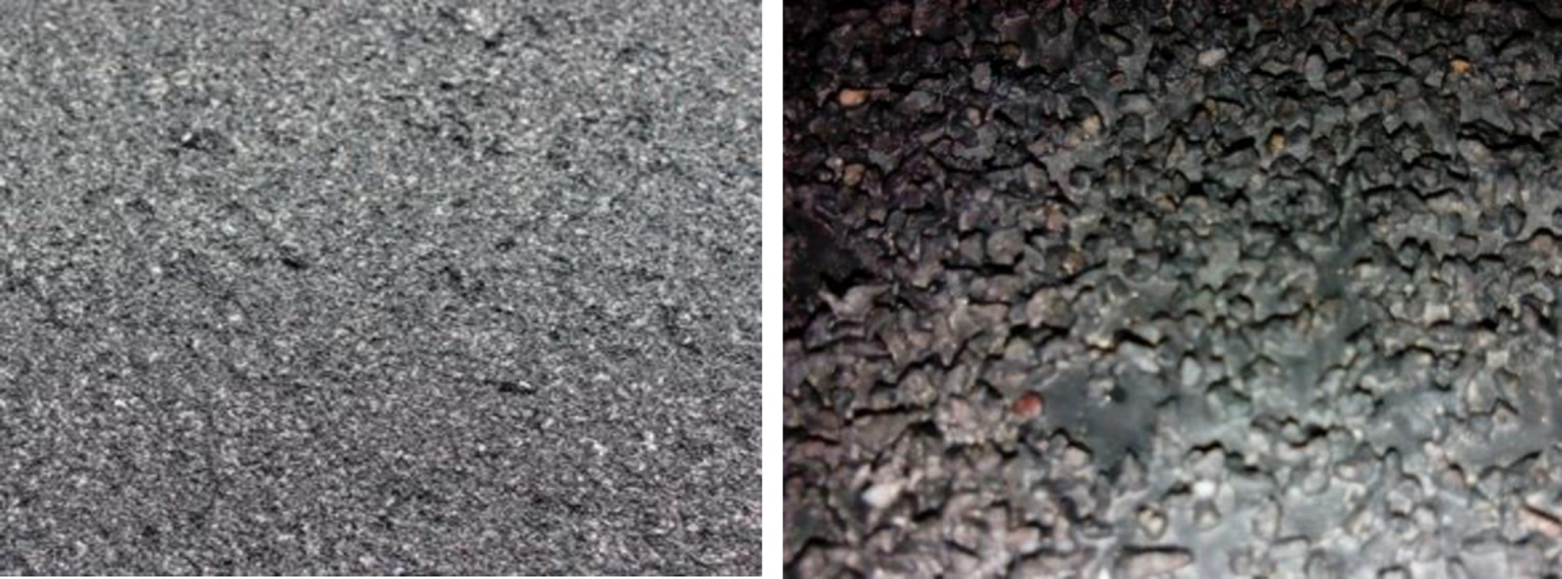
Specimen surface (left) and enlarged view (right).

The water-based epoxy resin modified emulsified asphalt was sprayed on the surface of the prepared concrete slab (smooth or rough) at a rate of 0.3kg/m^2^. Then four different anti-skid aggregates including limestone, basalt, silicon carbide, and natural river sand were evenly dispersed. The particle size of anti-skid aggregate was 0.3mm, 0.6mm, 0.3mm:0.6mm = 3:2, and the amount of anti-skid aggregate was 0.4kg/m^2^. The specimen was prepared after curing for 2h, as shown in Fig 6.

**Fig 7:**
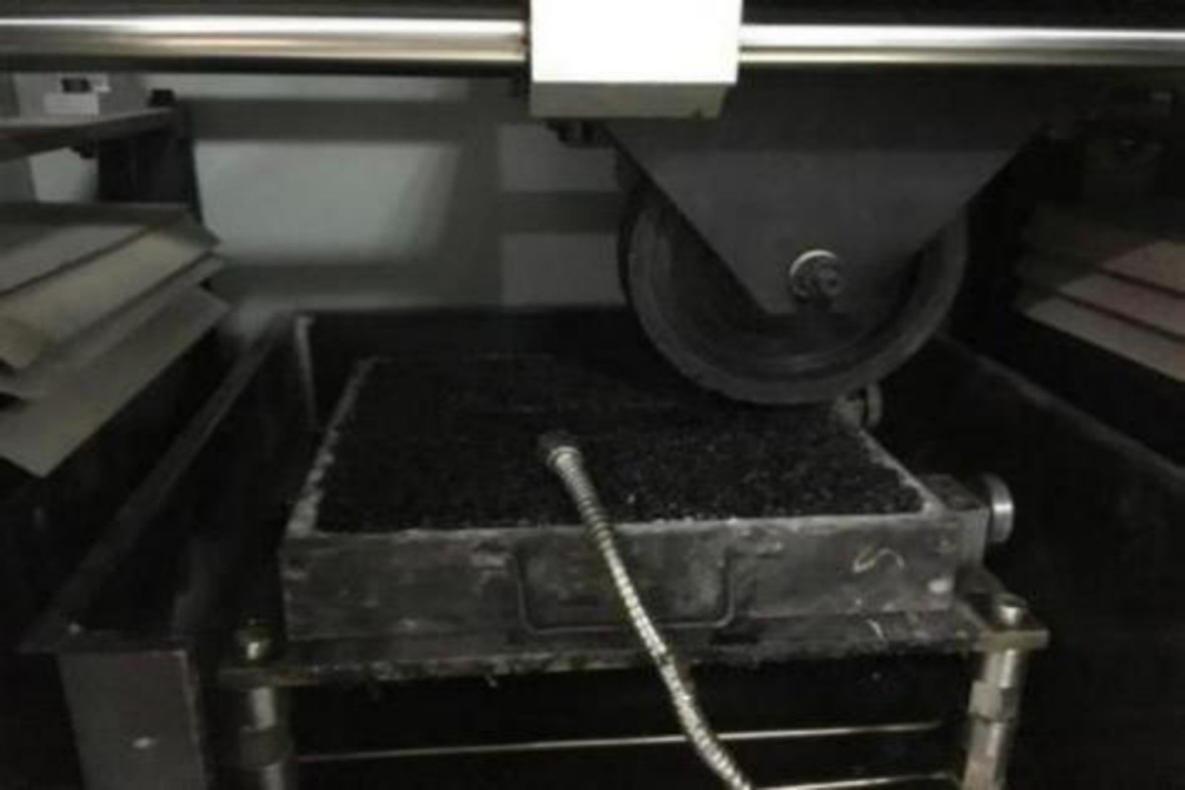
Rut wear test.

In order to study the long-term anti-skid performance of the treated surface, a rut tester was used to carry out the wear test, as shown in Fig 7. The wear times was set as 0, 20000, 40000, 60000, 80000 and 100000, and the BPN of the specimen was measured after each wear.

## 4 Experimental results and analysis

### 4.1 Performance of binder

#### 4.1.1 Evaporation residue content and three indicators

**Fig 8:**
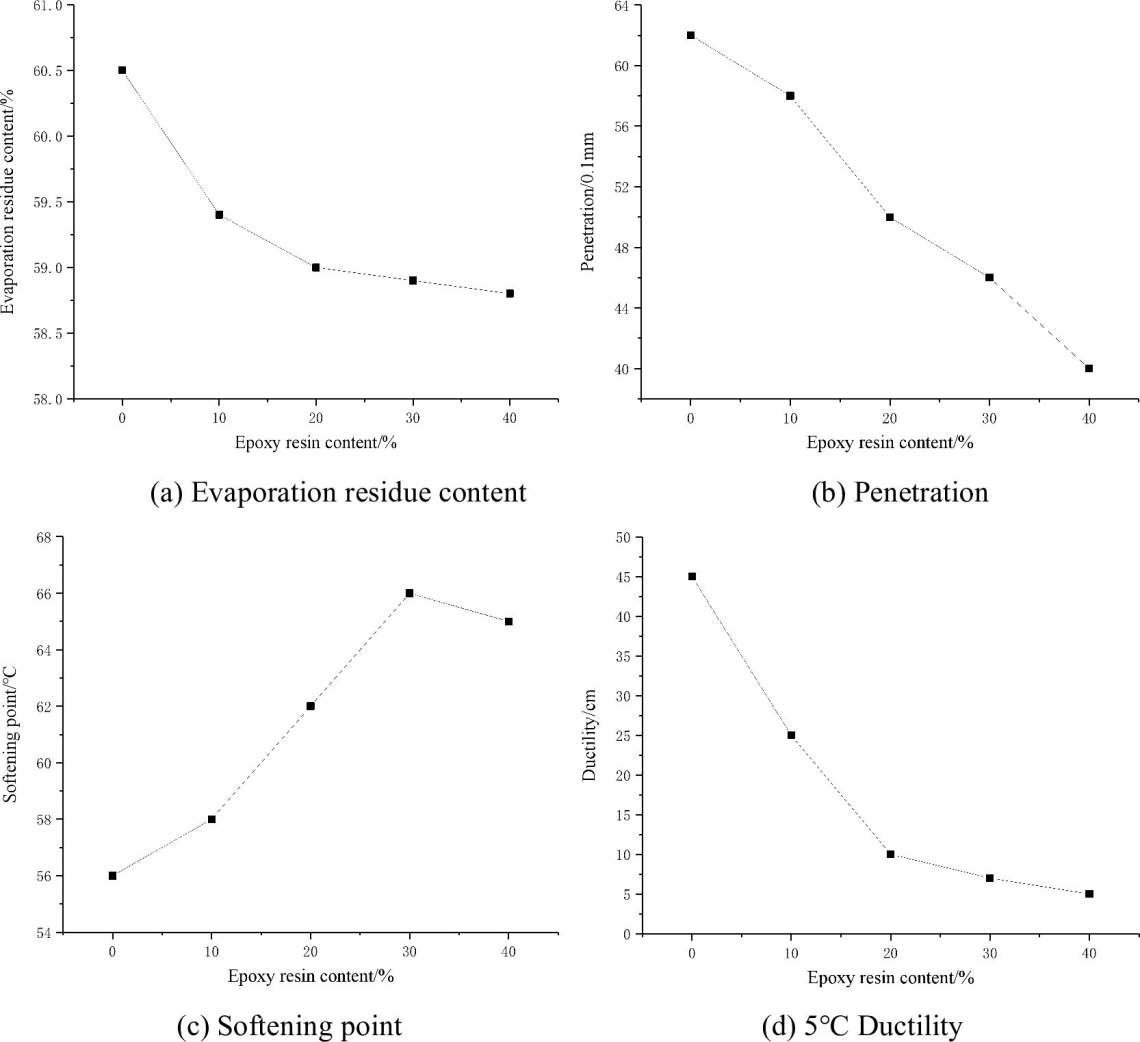
Evaporation residue test of water-based epoxy emulsified asphalt. (a) Evaporation residue content, (b) Penetration, (c) Softening point, and (d) 5°C Ductility.

The evaporation residue, penetration, softening point, and 5°C ductility test results of water-based epoxy emulsified asphalt are shown in Fig 8.

From Fig 8, it can be seen that the content, penetration, and softening point of the evaporated residue of emulsified asphalt decrease with the increase of the content of water-based epoxy resin. This is because the cross-linking reaction between epoxy resin and curing agent causes the asphalt to become hard and brittle. The more water-based epoxy resin is added, the more brittle the asphalt becomes. The softening point of evaporation residue first increased and then decreased with the increase of water-based epoxy resin content. When the water-based epoxy resin content was 30%, the softening point reached the maximum 66°C. Water-based epoxy resin can improve the high temperature performance of emulsified asphalt, but reduce the low temperature performance.

#### 4.1.2 Adhesion

**Fig 9:**
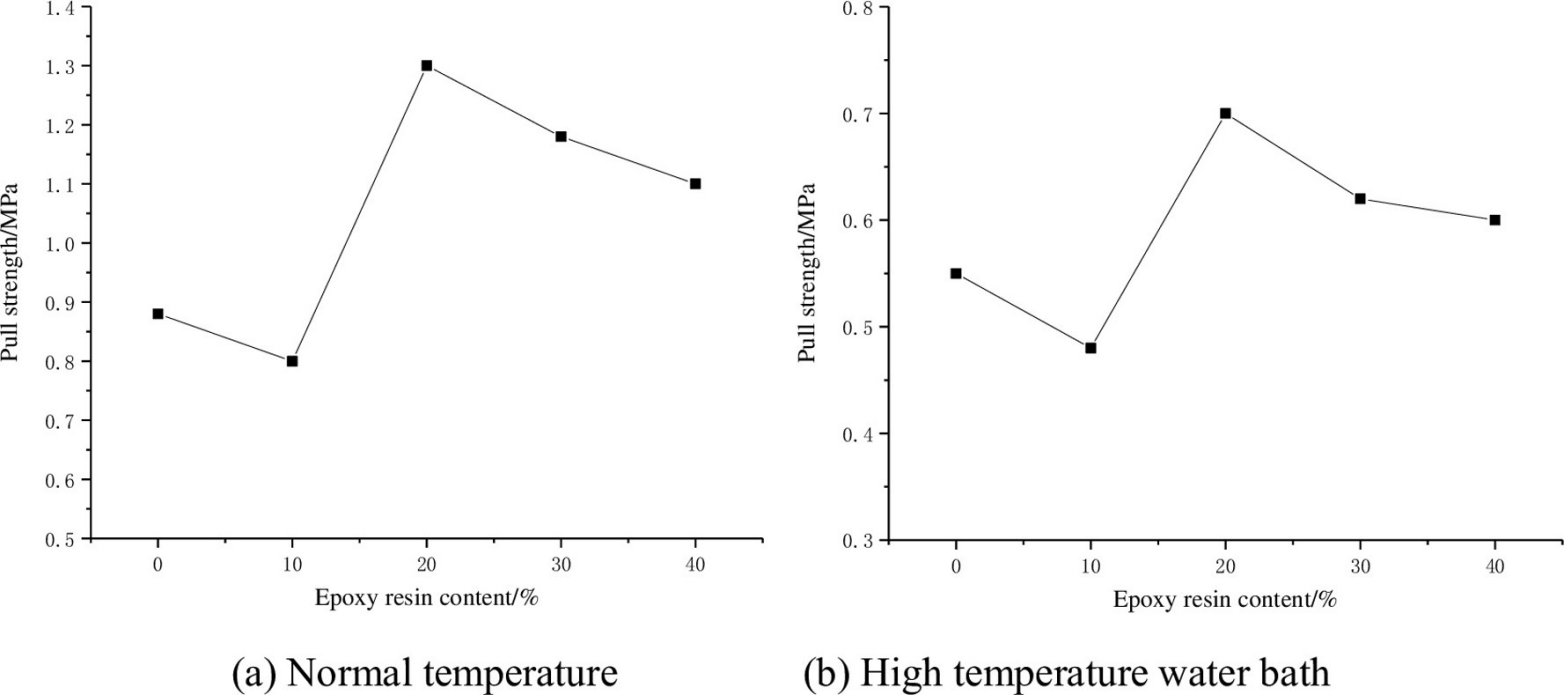
Adhesion test results. (a) Normal temperature and (b) High temperature water bath.

The adhesion test results under normal temperature and high temperature water bath are shown in Fig 9.

As can be seen from Fig 9A, when the water-based epoxy resin content is 10%, the adhesion is less than pure emulsified asphalt, because a small amount of epoxy resin can not form a network structure, and its function is equivalent to diluent. However, with the increase of water-based epoxy resin content, the adhesion of the binder increases first and then decreases, because the agglomeration phenomenon is easy to occur when the content of epoxy resin is too much. When the content of epoxy resin is 20%, the adhesion reaches the maximum of 1.3MPa. As can be seen from Fig 9B, after high temperature and water damage, the adhesion decreases significantly, and the maximum adhesion decreases 46% to 0.7MPa.

#### 4.1.3 Fluorescence microscopic test

**Fig 10:**
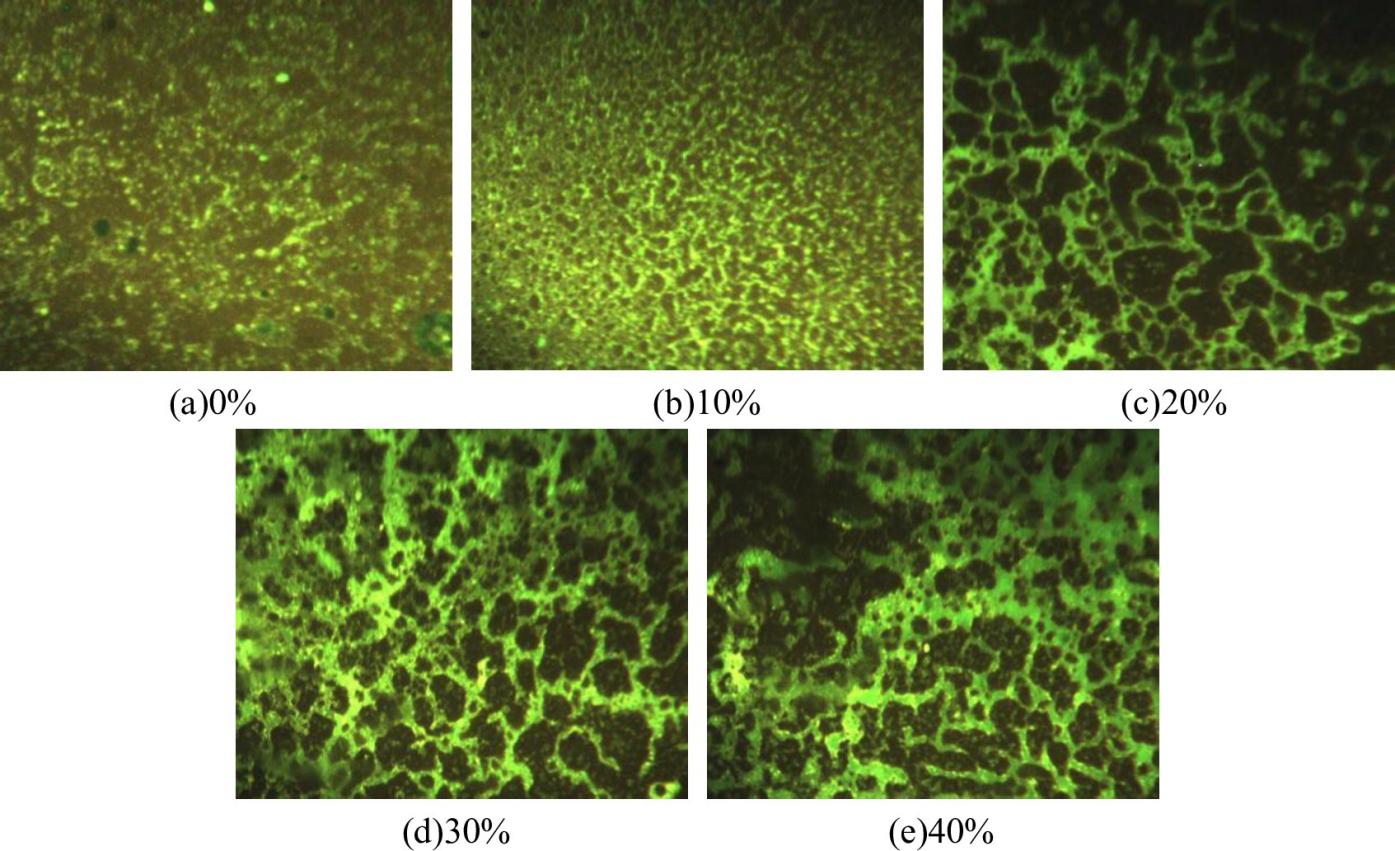
Fluorescence microscope image of water-based epoxy emulsified asphalt. (a) 0%, (b) 10%, (c) 20%, (d) 30%, and (e) 40%.

The fluorescence microscopy test results are shown in Fig 10.

As can be seen from Fig 10 that the network cross-linking begins to form when the content of water-based epoxy resin is 20%, and the network structure is more obvious when the content is 30%. When the content is above 40%, a small amount of aggregation occurs in the epoxy resin. It is recommended that the dosage of water-based epoxy resin is between 20 and 30%, so that the performance of modified emulsified asphalt is better.

### 4.2 Anti-skid performance of the roughened surfaces

#### 4.2.1 Initial skid resistance of roughened surfaces

**Fig 11:**
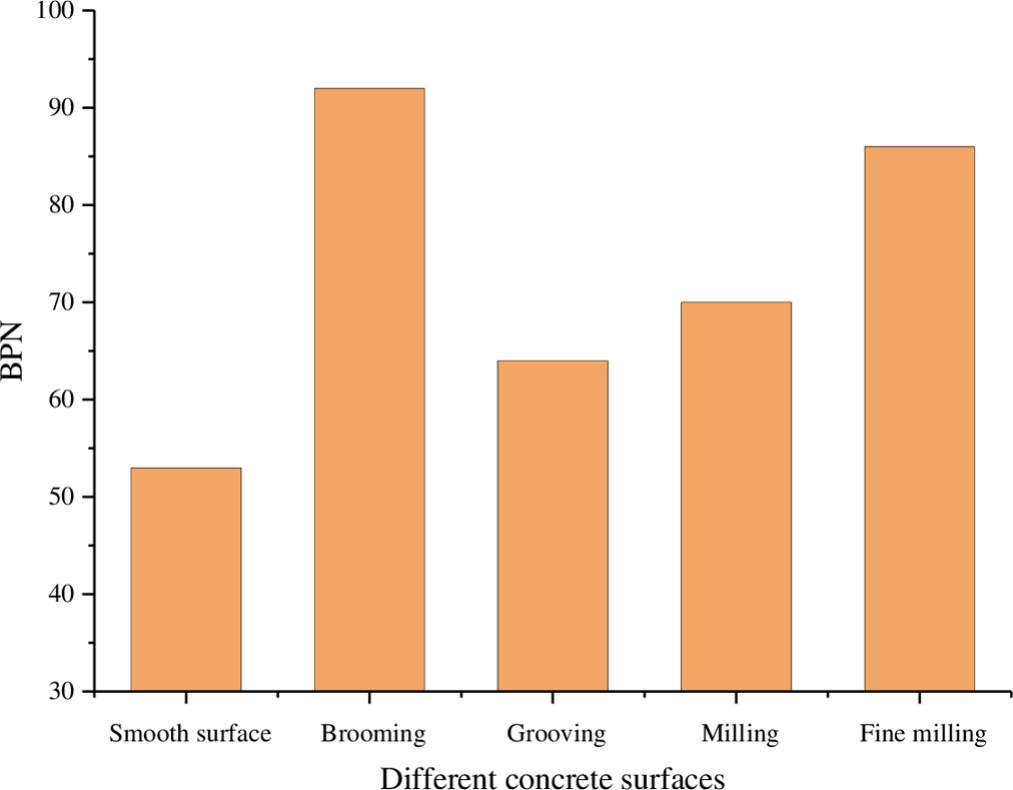
BPN of different treatment measures.

Anti-skid treatment measures of brooming, grooving, milling and fine milling were taken for concrete slabs, and the BPN of each treatment measure was tested respectively, with the results shown in Fig 11.

It can be seen from Fig 11 that the BPN of the untreated concrete slab is relatively low, around 53. After fine milling, the BPN is 86, which increases about 62%. After milling, the BPN is 70, which increases about 32%. After grooving, the BPN is 64, which increases about 20%. After brooming, the BPN is 92, which increases about 73%. All kinds of treatment measures can improve the anti-skid performance of concrete pavement rapidly, and the effect is brooming > fine milling > milling > grooving. However, the anti-skid durability of various roughened surfaces under the action of tire wear needs further study.

#### 4.2.2 Long-term skid resistance of roughened surfaces

**Fig 12:**
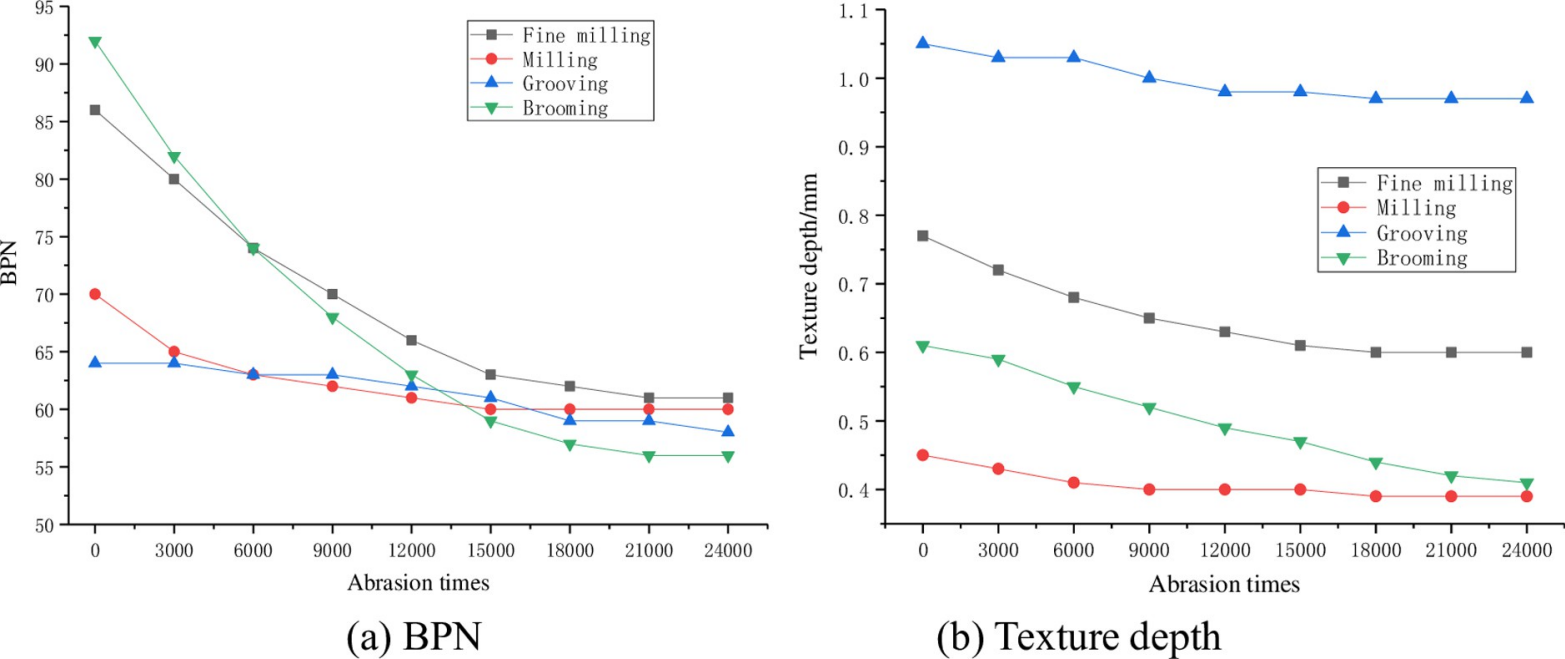
Anti-skid law of different roughening treatment measures. (a) BPN and (b) Texture depth.

The BPN and texture depth test results in the long-term wear process of the roughened surface are shown in Fig 12.

According to the wear test results in Fig 12A, it can be seen that: (1) the BPN of all kinds of roughened surfaces tend to decrease with the increase of the number of abrasion times, and the skid resistance after stability is fine milling (61) > milling (60) > grooving (58) > brooming (56). (2) With the increase of the number of wear, the BPN of the brooming and fine milling surface began to decline quickly, and then decreased slowly until stable. This is because the original texture surface is mainly mortar, easy to wear, resulting in a faster drop in BPN. After the stone slowly exposed, and stone is hard and not easy to be worn, the texture changes slowly, so the BPN drops slowly. Finally, the stone is completely exposed, and the texture is almost constant, so the BPN tends to be stable. After 21000 times of abrasion, BPN tended to be stable and decreased by about 29.1% and 39.1%, respectively. (3) Different from other roughening treatments, the BPN of concrete treated by milling attenuates slowly and becomes stable soon. This is because most of the floating slurry on the concrete upper layer has been milled out, and the BPN tends to stabilize after 12000 times of wear, about 60, which decreases 12.9% compared with the initial value. (4) For grooves, it can be found that the wear effect is not obvious, and the BPN is basically unchanged. As can be seen from the test results in Fig 12B that the texture depth of different roughening treatment measures decreases with the increase of the number of wear, and the structural depth after stabilization is in the order of grooving (0.97mm) > fine milling (0.60mm) > brooming (0.42mm) > milling (0.39mm).

### 4.3 Anti-skid performance of treated surface

#### 4.3.1 Influence of aggregate on skid resistance

**Fig 13:**
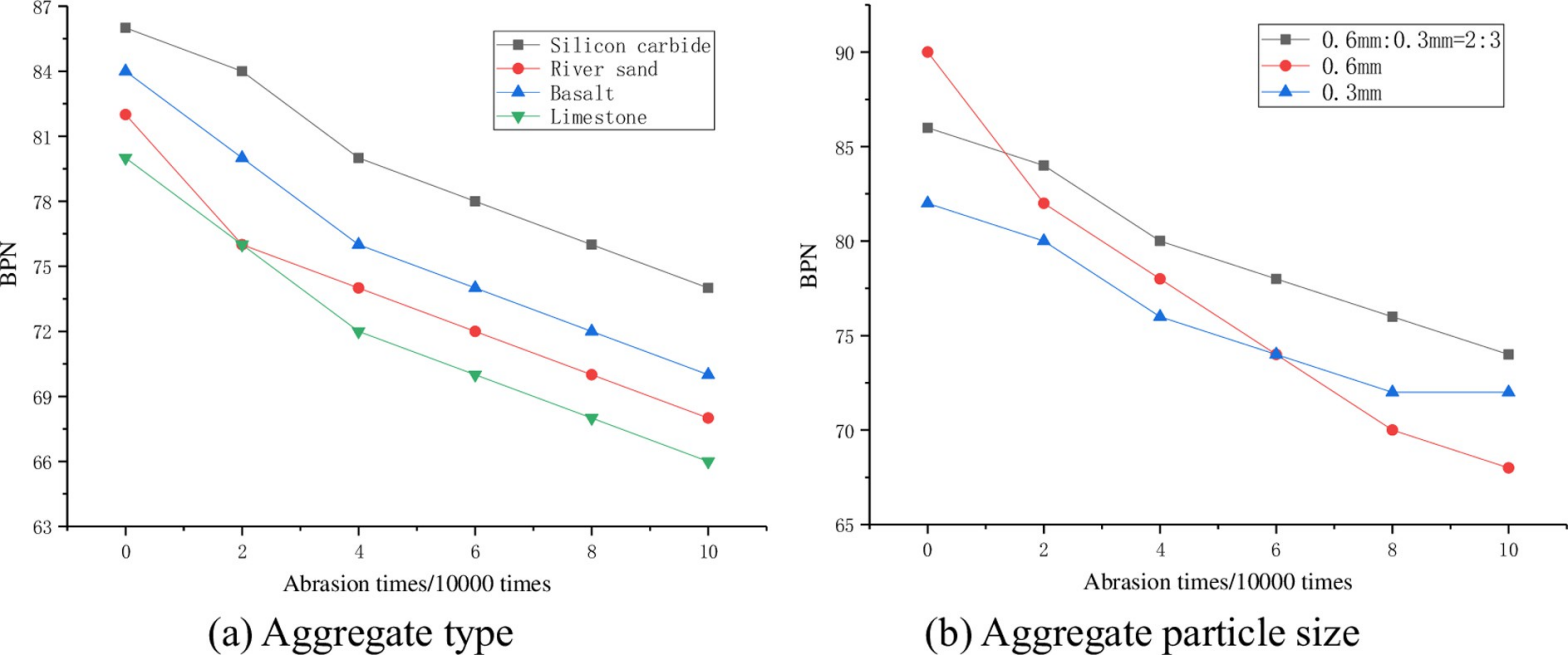
Influence of aggregate on the long-term skid resistance. (a) Aggregate type and (b) Aggregate particle size.

The influence of aggregate type and aggregate particle size on the long-term skid resistance of the treated surface is shown in Fig 13.

From the test results in Fig 13A, it can be seen that the order of anti-skid performance remains unchanged from beginning to end, with the order being: silicon carbide>basalt>natural river sand>limestone. From Fig 13B, it can be seen that for the initial anti-skid performance, the order is 0.6mm>0.6mm: 0.3mm = 2:3>0.3mm. For the stable anti-skid performance, the order is 0.6mm: 0.3mm = 2:3>0.3mm>0.6mm. Therefore, it is recommended to select silicon carbide as anti-skid aggregate, and the anti-skid effect is better when the particle size is 0.6mm: 0.3mm = 2:3.

#### 4.3.2 Influence of surface roughness on skid resistance

**Fig 14:**
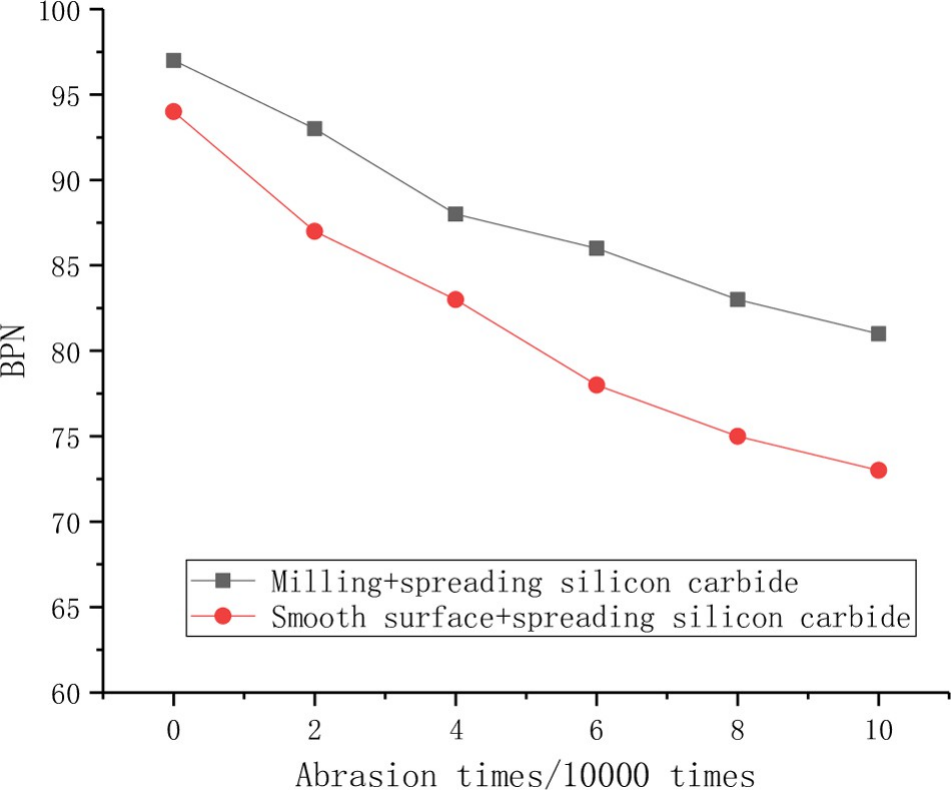
Influence of surface roughness on the long-term skid resistance.

The long-term anti-skid performance of smooth and rough concrete slabs after surface treatment is shown in Fig 14.

As can be seen from the test results in Fig 14, the anti-skid decay rate of melled concrete combined with surface treatment is more stable than that of smooth surface, which indicates that coarser concrete can provide more reliable and long-term anti-skid performance for pavement.

## 5 Test road paving and anti-skid performance testing

**Fig 15:**
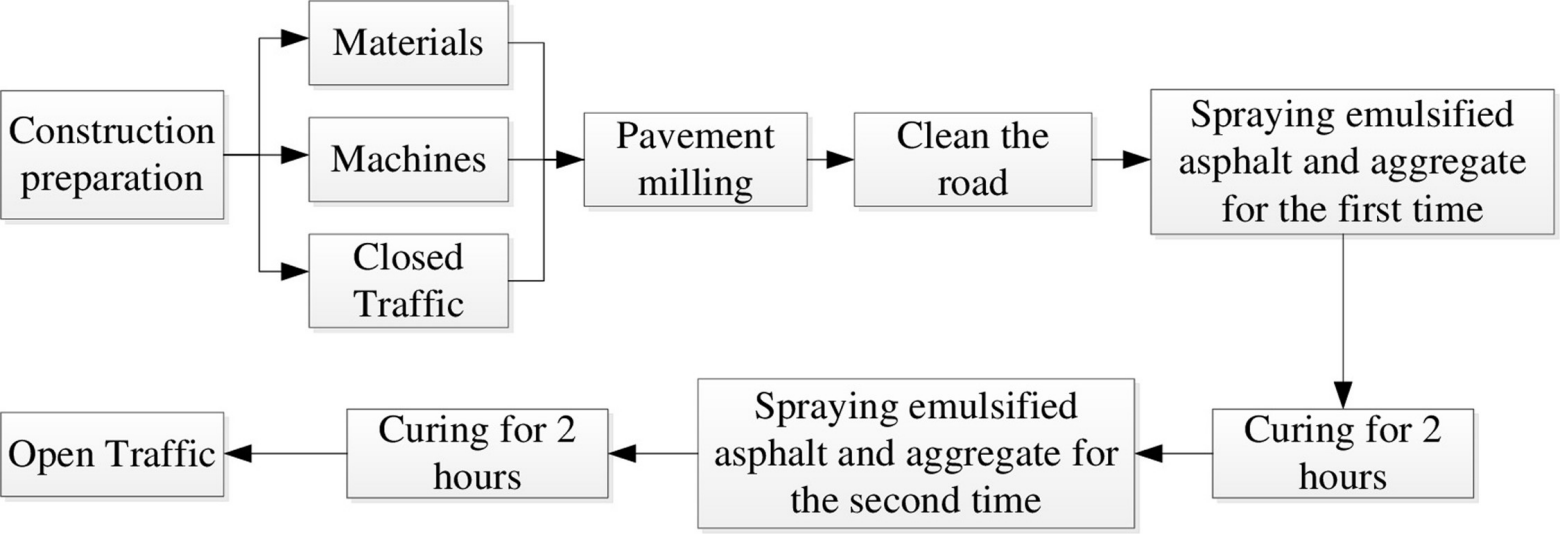
Construction process of anti slip surface treatment.

A concrete pavement of a highway tunnel in Sichuan Province, China was selected for anti-skid treatment test. The construction process is shown in Fig 15. The original road surface is roughened by milling, the amount of binder is 0.3 ∼ 0.4 kg/m^2^, and the amount of anti-skid aggregate is 0.3 ∼ 0.5 kg/m^2^.

**Fig 16:**
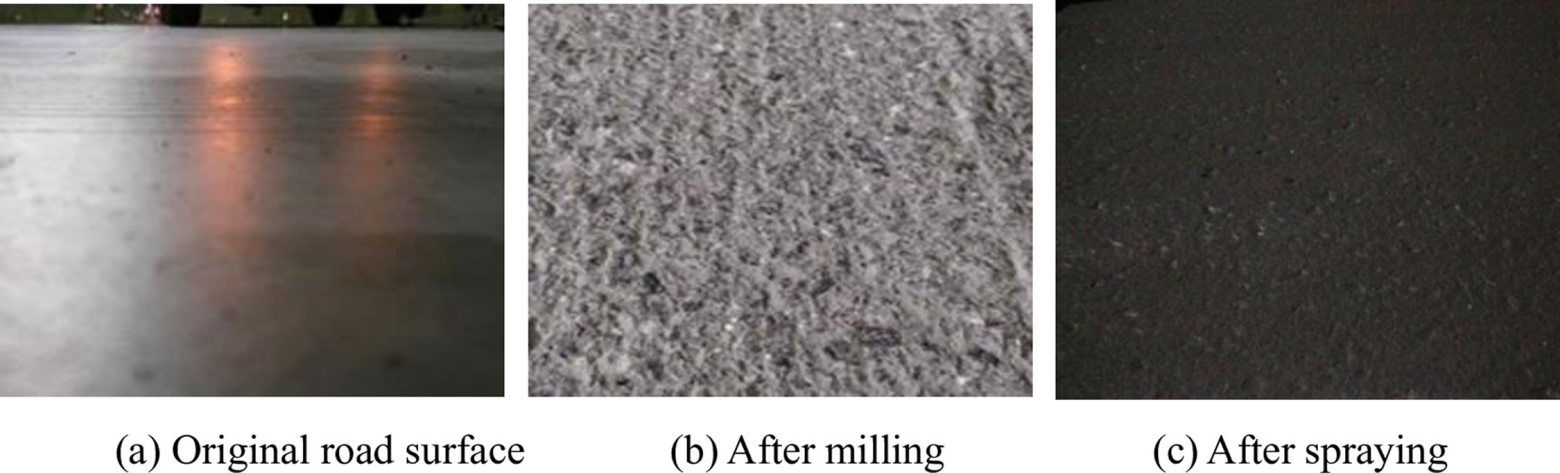
Comparison of road surface before and after treatment. (a) Original road surface, (b) After milling, and (c) After spraying.

The appearance of the tunnel pavement before and after treatment is shown in Fig 16.

**Fig 17:**
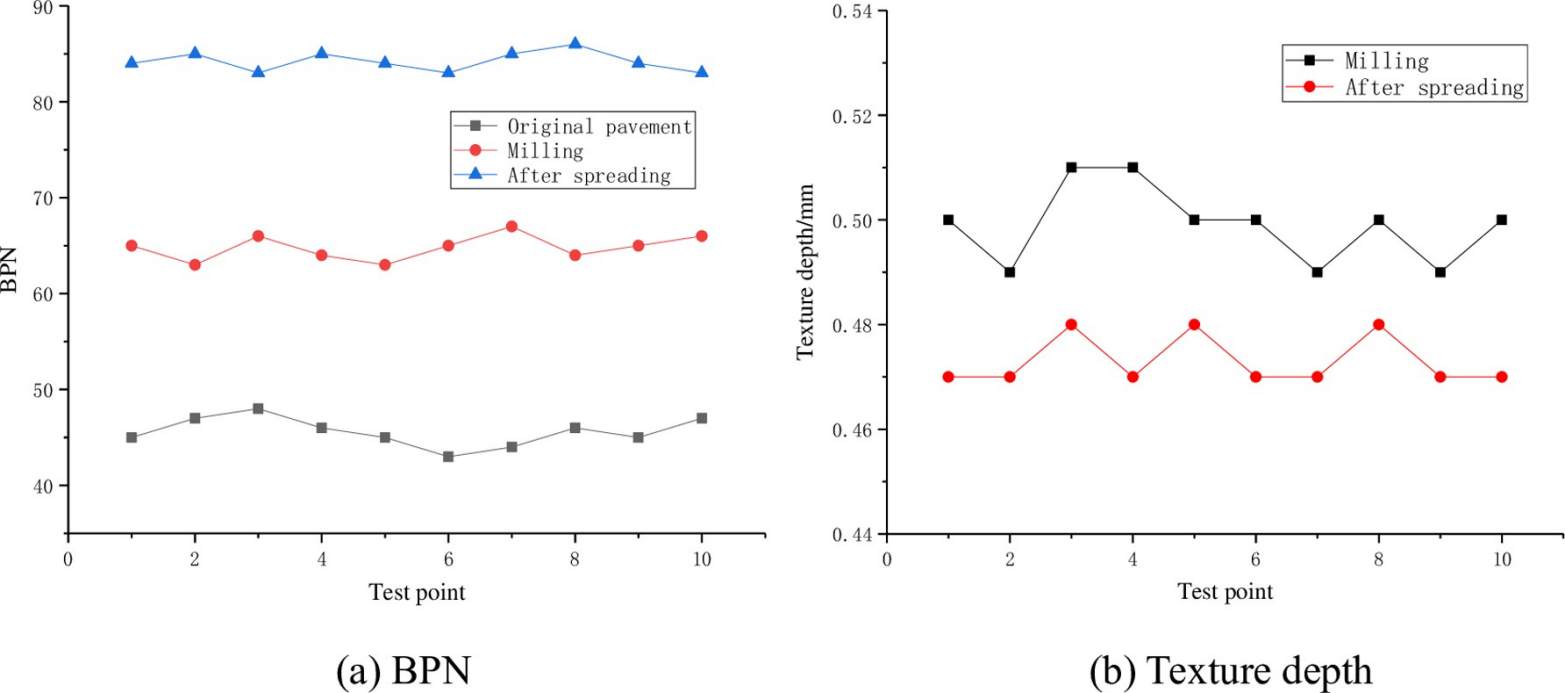
Anti-skid test results of tunnel pavement. (a) BPN and (b) Texture depth.

As can be seen from the comparison in Fig 16, the surface of the original concrete pavement is very smooth, and the appearance condition of the pavement has been well improved after milling and spraying of binder and aggregate. The test results of skid resistance before and after treatment are shown in Fig 17.

**Fig 18:**
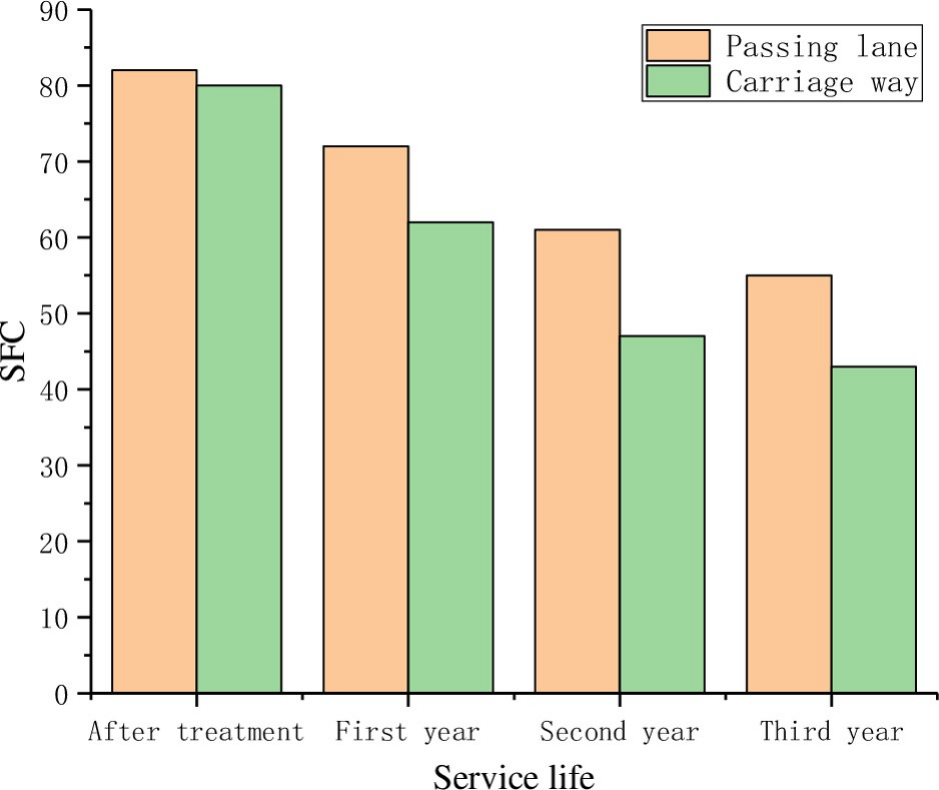
Skid resistance changes over time.

As can be seen from Fig 17A, both milling and surface treatment can improve the anti-skid performance of the original concrete pavement by about 40% and 80%, respectively. On the basis of milling, the surface treatment can improve the anti-skid performance of the pavement again, and its value is far beyond the requirements for anti-skid performance of the pavement in the specification. The concrete pavement in the original tunnel was remarkably polished, and its texture depth could not be measured by the sand laying method. Therefore, the texture depth of the pavement after milling and surface treatment was only tested, and the results were shown in Fig 17B. The texture depth of pavement was improved after treatment. Compared with milling, the structural depth after spreading the binder and aggregat is reduced but not significantly. The anti-skid performance of the tunnel pavement carriage way and passing lane after treatment was further tracked and detected, and the results were shown in Fig 18.

As can be seen from the results in Fig 18, with the increase of service life, the transverse force friction coefficient SFC of the road surface decreases. Due to the larger traffic volume and more serious road wear, the SFC of the passing lane is larger than that of the carriage lane. After three years of operation, the anti-skid performance of the road surface still maintains a high level, which indicates the feasibility of anti-skid treatment technology.

## 6 Conclusions

In order to rapidly improve the skid resistance of tunnel concrete pavement, treatment measures of concrete surface roughening + spraying epoxy emulsified asphalt + spreading anti-skid aggregate were proposed in this study, and the optimal combination was determined through long-term abrasion test. The main conclusions are as follows:

1. The addition of water-based epoxy resin will improve the high-temperature performance of emulsified asphalt and increase its adhesion to the surface of concrete road, and the recommended dosage is 20%∼30%;
2. Four kinds of surface roughening methods, including fine milling, milling, grooving and brooming, can increase the skid resistance of concrete, and the skid durability of fine milling and milling is better than grooving and brooming;
3. It is recommended to select silicon carbide as anti-skid aggregate, and the anti-skid effect is better when the particle size is 0.6mm: 0.3mm = 2:3.
4. The method of fine milling + spraying water-based epoxy emulsified asphalt + spreading silicon carbide can effectively improve the skid resistance of the tunnel concrete pavement, and the feasibility of the scheme has been verified by the test road.

## References

[1] Toraldo E. Comparative laboratory investigation into pavement materials for road tunnels. Road Materials And Pavement Design, 2013,14(2):310–324. doi: 10.1080/14680629.2013.794366

[2] Fang JM, Tu JS, Wu KM. Analysis of Skid Resistance and Noise Characteristics for Varieties of Concrete Pavement. Advances in Materials Science And Eegineering, 2020. doi: 10.1155/2020/7427314

[3] Yang JG, Xie YL, Zhang X, Ma W. Reliability Analysis on Pavement Skid-Resistant Performance in Expressway Tunnels. Journal of Southwest University, 2009,31(11):145–149. (in Chinese)

[4] Ding YJ, Li DN, Huang MX, et al. Study on the influence of skid resistance on traffic safety of highway with a high ratio of bridges and tunnels. Transportation Safety And Environment, 3(4). doi: 10.1093/tse/tdab025

[5] Cai ZS, Fan JW, Ma T, et al. Aggregate-Exposing Operation Parameters, Laboratory and Road Performances of Exposed-Aggregate Concrete Pavement Applied in Long Tunnel. Journal of Testing And Evaluation, 2022. doi: 10.1520/JTE20220054

[6] Marcobal JR, Salado F, Flintsch G. Evaluation of Various Surface Cleaning Techniques Inside Tunnels on Pavement Skid Resistance. Materials, 2021,14(19). doi: 10.3390/ma14195660 34640057 PMC8510209

[7] Li B, Kang HW, Zhang ZW. Comparison of Skid Resistance and Noise between Transverse and Longitudinal Grooving Pavements in Newly Constructed Concrete Pavement. Advanced Materials Research, 2012, 446–449:2637–2640. doi: 10.4028/www.scientific.net/AMR.446-449.2637

[8] Zheng ML, Tian YJ, Wang XP, Peng P. Research on Grooved Concrete Pavement Based on the Durability of Its Anti-Skid Performance. Applied Sciences, 2018, 8(6):891. doi: 10.3390/app8060891

[9] Hui B, Liang HM, Li SQ, et al. Quality control of micro-milling treatment on tunnel concrete pavement using 3D range data. International Journal Of Pavement Eegineering, 23(5):1612–1621. doi: 10.1080/10298436.2020.1815743

[10] Ren WY, Han S, Li J, Liu MM. Investigation of the relative abrasion resistance of concrete pavement with chip-sprinkled surfaces. Wear, 32:95–101. doi: 10.1016/j.wear.2017.04.011

[11] Cui PD, Wu SP, Xiao Y, et al. Enhancement mechanism of skid resistance in preventive maintenance of asphalt pavement by steel slag based on micro-surfacing. Construction And Building Materials, 2020,239. doi: 10.1016/j.conbuildmat.2019.117870

[12] Kandhal PS, Lockett L. Construction and performance of ultrathin asphalt friction course. Symposium on Flexible Pavement Rehabilitation and Maintenance, 1998,(1348):81–95. doi: 10.1520/STP12853S

[13] Hong B, Lu GY, Gao JL, et al. Green tunnel pavement: Polyurethane ultra-thin friction course and its performance characterization. Journal of Cleaner Production, 2021,289. doi: 10.1016/j.jclepro.2020.125131

[14] Yu JM, Chen YL, Wei XP, et al. Performance Evaluation of Ultra-Thin Wearing Course with Different Polymer Modified Asphalt Binders. Polymers, 2022,14(16). doi: 10.3390/polym14163235 36015492 PMC9416601

[15] Liu ZM, Luo S, Quan X, et al. Laboratory evaluation of performance of porous ultra-thin overlay. Construction And Building Materials, 2019,(204):28–40. doi: 10.1016/j.conbuildmat.2019.01.147

[16] Ahammed MA, Tighe SL. Concrete pavement surface textures and multivariables frictional performance analysis: a North American case study. Canadian Journal of Civil Engineering, 2008,35(7):727–738. doi: 10.1139/L08-025

[17] Chen Y, Wang KJ, Zhou WF. Evaluation of surface textures and skid resistance of pervious concrete pavement. Journal of Central South University, 2013,20(2):520–527. doi: 10.1007/s11771-013-1514-y

[18] Kane M, Rado Z, Timmons A. Exploring the texture-friction relationship: from texture empirical decomposition to pavement friction. International Journal of Pavement Eegineering, 16(10):919–928. doi: 10.1080/10298436.2014.972956

[19] Yang XY, Lu B, Jing QS, et al. Macrotexture deterioration for micromilled tunnel concrete pavement using 3D laser data. Measurement Science Aad Technology, 2023,34(6). doi: 10.1088/1361-6501/acbc3d

[20] Sadowski L, Stefaniuk D. The effect of surface treatment on the microstructure of the skin of concrete. Applied Surface Science, 427:934–941. doi: 10.1016/j.apsusc.2017.09.078

[21] Gierasimiuk P, Wasilewska M, Gardziejczyk W. A Comparative Study on Skid Resistance of Concrete Pavements Differing in Texturing Technique. Materials, 2021, 14(1). doi: 10.3390/ma14010178 33401456 PMC7794823

